# Incidence, risk factors and prediction of post-operative acute kidney injury following cardiac surgery for active infective endocarditis: an observational study

**DOI:** 10.1186/cc13041

**Published:** 2013-10-04

**Authors:** Matthieu Legrand, Romain Pirracchio, Anne Rosa, Maya L Petersen, Mark Van der Laan, Jean-Noël Fabiani, Marie-paule Fernandez-gerlinger, Isabelle Podglajen, Denis Safran, Bernard Cholley, Jean-Luc Mainardi

**Affiliations:** 1 Department of Anesthesiology and Critical Care, Hôpital Européen Georges Pompidou Assistance Publique-Hopitaux de Paris; Université Paris Descartes, Sorbonne Paris Cité, Paris, France; 2 Unité INSERM UMR-S942, Hôpital Lariboisière, 2, Rue Ambroise Paré, Paris 75010, France; 3 Unité INSERM UMR-S717, Hôpital Saint Louis, 1, Avenue Claude-Vellefaux, Paris 75010, France; 4 School of Public Health, Division of Biostatistics, University of California at Berkeley, Berkeley CA, USA; 5 Vascular and Heart Surgery, Hôpital Européen Georges Pompidou, Assistance Publique- Hopitaux de Paris; Université Paris Descartes, Sorbonne Paris Cité, Paris, France; 6 Laboratory of microbiology, Hôpital Européen Georges Pompidou, Assistance Publique- Hopitaux de Paris; Université Paris Descartes, Sorbonne Paris Cité, Paris, France; 7 Department of Anesthesiology and Critical Care and Burn Unit, Hôpitaux Universitaires Saint-Louis-Lariboisière, Assistance Publique - Hôpitaux de Paris, Université Paris 7 Denis Diderot, 1 avenue Claude Vellefaux, Paris Cedex 10, France

## Abstract

**Introduction:**

Cardiac surgery is frequently needed in patients with infective endocarditis (IE). Acute kidney injury (AKI) often complicates IE and is associated with poor outcomes. The purpose of the study was to determine the risk factors for post-operative AKI in patients operated on for IE.

**Methods:**

A retrospective, non-interventional study of prospectively collected data (2000–2010) included patients with IE and cardiac surgery with cardio-pulmonary bypass. The primary outcome was post-operative AKI, defined as the development of AKI or progression of AKI based on the acute kidney injury network (AKIN) definition. We used ensemble machine learning (“Super Learning”) to develop a predictor of AKI based on potential risk factors, and evaluated its performance using V-fold cross validation. We identified clinically important predictors among a set of risk factors using Targeted Maximum Likelihood Estimation.

**Results:**

202 patients were included, of which 120 (59%) experienced a post-operative AKI. 65 (32.2%) patients presented an AKI before surgery while 91 (45%) presented a progression of AKI in the post-operative period. 20 patients (9.9%) required a renal replacement therapy during the post-operative ICU stay and 30 (14.8%) died during their hospital stay. The following variables were found to be significantly associated with renal function impairment, after adjustment for other risk factors: multiple surgery (OR: 4.16, 95% CI: 2.98-5.80, p<0.001), pre-operative anemia (OR: 1.89, 95% CI: 1.34-2.66, p<0.001), transfusion requirement during surgery (OR: 2.38, 95% CI: 1.55-3.63, p<0.001), and the use of vancomycin (OR: 2.63, 95% CI: 2.07-3.34, p<0.001), aminoglycosides (OR: 1.44, 95% CI: 1.13-1.83, p=0.004) or contrast iodine (OR: 1.70, 95% CI: 1.37-2.12, p<0.001). Post-operative but not pre-operative AKI was associated with hospital mortality.

**Conclusions:**

Post-operative AKI following cardiopulmonary bypass for IE results from additive hits to the kidney. We identified several potentially modifiable risk factors such as treatment with vancomycin or aminoglycosides or pre-operative anemia.

## Introduction

Early surgical treatment has been suggested to improve the outcome of patients with infective endocarditis (IE) [1]. Acute kidney injury (AKI) is a common complication following cardiac surgery, occurring in 5 to 20% of the patients [2-5]. It has been associated with increased mortality [3,6,7]. Furthermore, AKI has been reported to be associated with poor prognosis in patients with acute IE [8-10]. There is therefore a need to prevent episodes of post-operative AKI, which may improve the outcome.

Patients with IE are typically at risk of kidney injury and their renal function might further be compromised because of cumulative injuries occurring before, during and after surgery. However, there are limited data on the prognosis of AKI in patients with IE undergoing cardiac surgery, and particularly, the risk factors favoring post-operative worsening of renal function in this context are not well-described. The aim of the present study was to describe the incidence of AKI patients undergoing operation for an acute episode of endocarditis, and to identify the risk factors for post-operative AKI or worsening of renal function in these patients.

## Materials and methods

### Patients

Between January 2000 and December 2010, all consecutive patients admitted to the Hôpital Européen Georges Pompidou (Paris, France), an 800-bed university hospital with a dedicated infectious unit specializing in treatment of patients with IE, with the diagnosis of IE according to the modified Duke criteria [11] and who underwent cardiac surgery with cardiopulmonary bypass were included. The study was approved by our local ethical committee (CEERB Paris Nord) and no consent was needed. The indications for surgery were refractory congestive heart failure, endocarditis-related dysfunction of native or prosthetic valve, uncontrolled infection, perivalvular extension of infection, systemic embolic episode or large vegetation. The patients who died within the first 24 hours following surgery and those who needed pre-operative renal replacement therapy (RRT) for any reason were excluded from the analysis.

### Data collection

Epidemiological, clinical and biological data were prospectively collected using a standardized case report form. Clinical and epidemiological data collected are presented in Table 1.

**Table 1:** Patients’ characteristics

	**All patients (n = 202)**	**No post-operative AKI (n = 82)**	**Post-operative AKI (n = 120)**	***P*-value**
Age, years, median (IQR)	42 (28 to 59)	50 (37 to 66)	36 (27 to 51)	<0.001
Gender, male, n (%)	134 (66.3)	49 (59.8)	85 (70.8)	0.14
**Comorbidities, n (%)**				
COPD	9 (4.4)	2 (2.4)	7 (5.8)	0.42
Diabetes mellitus	22 (10.9)	7 (8.5)	15 (12.5)	0.63
Hypertension	60 (29.7)	22 (26.8)	38 (31.7)	0.56
Heart failure	13 (6.4)	3 (3.7)	10 (8.3)	0.30
CAD	11 (5.4)	3 (3.7)	8 (6.7)	0.54
Autoimmune disease	8 (4.0)	4 (4.9)	4 (3.3)	0.85
Liver disease	17 (8.4)	5 (6.1)	12 (10.0)	0.47
Cancer	17 (8.4)	6 (7.3)	11 (9.2)	0.84
**Pre-operative condition**				
Mechanical ventilation, n (%)	16 (7.9)	2 (2.4)	14 (11.7)	0.03
Cathecolamine use, n (%)	18 (8.9)	4 (4.9)	14 (11.7)	0.16
Norepinephrine, (%)	9 (4.4)	1 (1.2)	8 (6.7)	0.13
Epinephrine, n (%)	3 (1.5)	0 (0)	3 (2.5)	0.39
Dobutamine, n (%)	9 (4.4)	4 (4.9)	5 (4.2)	1
% LVEF, median (IQR)	65 (60 to 70)	65 (60 to 70)	65 (58 to 70)	0.10
Baseline serum creatinine, μmol/l, median (IQR)	83 (75 to 100)	78 (62 to 86)	90 (80 to 100)	<0.001
Pre-operative serum creatinine, μmol/l, median (IQR)	100 (76 to 132)	77 (62 to 100)	116 (91 to 157)	<0.001
Bilirubin, mg/ml, median (IQR)	13 (10 to 20)	12 (9 to 15)	14 (11 to 22)	0.02
Platelet count, G/l, median (IQR)	249 (187 to 319)	261 (205 to 324)	244 (173 to 307)	0.06
CRP, median (IQR)	51 (11 to 87)	46 (17 to 106)	67 (30 to 159)	0.10
Hemoglobin, g/dl, median (IQR)	10.8 (9.5 to 11.9)	11 .3 (10.3 to 12.3)	10.3 (9.1 to 11.4)	<0.001
**Pre-operative nephrotoxic agents, n (%)**				
Contrast agent	94 (46.5)	36 (43.9)	58 (48.3)	0.63
Vancomycin	36 (17.8)	6 (7.3)	30 (25.0)	0.002
Aminoglycoside	119 (58.9)	45 (54.9)	74 (61.7)	0.41
**Surgery**				
Emergency surgery, n (%)	27 (13.4)	9 (11.0)	18 (15.0)	0.54
CPB duration, minutes, median (IQR)	118 (85 to 163)	100 (76 to 142)	118 (95 to 1800	0.004
Aortic clamping duration, minutes, median (IQR)	89 (65 to 121)	76 (59 to 100)	90 (74 to 132)	0.002
Circulatory arrest, n (%)	5 (2.5)	2 (2.4)	3 (2.5)	1

AKI, acute kidney injury; COPD, chronic obstructive pulmonary disease; CAD, coronary artery disease; LVEF, left ventricular ejection fraction; CPB, cardiopulmonary bypass; CRP, C reactive protein.

### Outcome measure

The main goal of the present study was to identify the risk factors for worsening of renal function during the peri-operative period in patients operated on for an acute episode of IE. Therefore, the primary outcome measure was post-operative AKI, defined as the development or the progression of AKI during the seven days following cardiac surgery with cardiopulmonary bypass. Explicitly, post-operative AKI was defined as (1) the development of AKI in patients with normal renal function before surgery, or (2) the progression of AKI when AKI was already present before surgery.

We used the acute kidney injury network (AKIN) classification [12] to define and stage AKI. AKI was defined as an increase of serum creatinine >50% or >26 μmol/l from baseline (stage 1 of the AKIN definition). In patients with pre-operative AKI, progression of AKI was defined as a post-operative increase in the stage of AKI or a need for RRT during the 7 days following surgery. Baseline serum creatinine was retrieved from the blood sample obtained before hospital admission, when available. When the baseline creatinine level was not available, the lowest serum creatinine level measured during the hospital stay was used if the glomerular filtration rate (GFR) was ≥75 mL/minute/1.73 m^2^. In the remaining cases, serum creatinine was estimated by the modification of diet in renal disease (MDRD) equation using a GFR of 75 mL/minute/1.73 m^2^.

### Statistical analysis

We first developed a prediction model for post-operative AKI based on the approach known as super learning, and then identified the most important risk factors using targeted maximum likelihood estimation (TMLE).

#### Super learner

The discrete super learner has been proposed by Dudoit and Van der Laan *et al*. [13] as a generalization of the stacking algorithms [14], to choose the optimal regression algorithm among a set of candidates. The underlying selection strategy relies on the choice of a loss function, which aims at evaluating the gap between the actual and the predicted outcomes for each candidate. The comparison between candidates relies on V-fold cross-validation: the dataset is divided into *V* mutually exclusive and exhaustive subsets of nearly equal size. At each *V* step of the procedure, one of the *V* sets serves as a validation set, while the others play the role of training sets for each candidate algorithm. Observations in the training set are used to construct the estimators, and observations in the validation set are used to assess the performance of the estimators, the so-called risk, on the basis of the chosen loss function (L2 or squared error in the present study). At the end of the procedure, each set has served both as the training and validation sample. For each candidate algorithm, the super learner then averages the *V* estimated risks over the *V* validation sets, resulting in the so-called cross-validated risk, for each candidate algorithm. Based on their cross-validated risks, the candidate estimators can be ranked and the optimal learner is finally applied to the entire dataset. Finally, a weighted linear combination of the candidate learners is used to build a new estimator, the so-called super learner estimator [15].

We investigated the following algorithms: generalized additive model, generalized linear model, stepwise regression (forward and based on the Akaike information criterion (AIC)), polynomial linear model, random forest, neural network, Bayesian generalized linear model, elastic-net regularized generalized linear model, polynomial spline regression and gradient boosting. The set of explanatory variables encompassed age, gender, pre-existing comorbidities (history of heart failure, chronic respiratory failure, hypertension, diabetes, coronary artery disease, autoimmune disease, cancer, liver disease or previous endocarditis), health status before surgery (presence of shock, systemic emboli, New York Heart Association (NYHA) classification, hemoglobin levels, baseline creatinine levels, or need for mechanical ventilation), characteristics of the infection (cardiac valve involvement, multiple valve infection, infection of native versus prosthetic valve, presence of pacemaker infection, or presence of positive blood cultures), characteristics of the surgery (emergency surgery within 24 h of admission, cardiopulmonary bypass duration, aortic clamping duration, circulatory arrest, implantation of a bioprothesis versus mechanical valve, valvular plasty, aortic tube, multiple surgery), use of nephrotoxic agents (contrast agents, aminoglycoside, or vancomycin) within 48 h prior to surgery and the following interactions terms: vancomycin-aminoglycoside, vancomycin-contrast, aminoglycoside-contrast. Pre-operative hemoglobin level before surgery was assessed on the day of surgery, multiple surgery was defined as a least one revision surgery. The risks associated with the different estimators were evaluated using the area under the receiver operating curve (AUROC), based on cross-validation.

#### Targeted maximum likelihood estimation for variable importance measure

In order to identify the most important risk factors for post-operative AKI, we used the TMLE approach. The variables considered as potential risk factors were: age; gender; pre-existing comorbidities (history of heart failure, chronic respiratory failure, hypertension, diabetes, coronary artery disease, autoimmune disease, cancer, liver disease or previous endocarditis); health status before surgery (presence of shock, systemic emboli, NYHA classification, hemoglobin levels, baseline creatinine levels, or need for mechanical ventilation); characteristics of the infection (cardiac valve involvement, multiple valve infection, infection of native versus prosthetic valve, presence of pace maker infection, or presence of positive blood cultures); characteristics of the surgery (emergency surgery within 24 h of admission, cardiopulmonary bypass duration, aortic clamping duration, circulatory arrest, implantation of a bioprothesis versus mechanical valve, valvular plasty, aortic tube, or multiple surgery); transfusion requirement during surgery; the use of nephrotoxic agents (contrast agents, aminoglycoside, or vancomycin) within 48 hours prior to surgery; and the following interactions terms: vancomycin-aminoglycoside, vancomycin-contrast, aminoglycoside-contrast.

Continuous variables that were considered as target parameters were dichotomized as follows: age < or ≥65 y, NYHA classification < or ≥III, baseline creatinine ≤ or >106 μmol/l, baseline hemoglobin < or ≥10 g/dl, extracorporeal circulation duration ≤ or >120 minutes, aortic clamping duration ≤ or >90 minutes. These thresholds were chosen according to the risk factors for AKI post-cardiac surgery, as identified by Palomba *et al*. [2]. For each potential risk factor, a variable importance measure was obtained adjusted after for all other risk factors [16-18]. The parameter targeted for measuring the importance of each covariate was the average difference in the outcome given the level of the candidate risk factor, after adjusting for all other covariates.

TMLE is a two-step procedure: first, running an initial regression to fit the expected value of the outcome given the covariate of interest (that is, the candidate risk factor in our situation) and adjusting for all other covariates. This first step may involve super learning; second, updating the initial regression relying on a fit of the propensity score, in order to obtain an optimal bias-variance trade-off for the parameter of interest. This procedure is repeated for each target parameter. Standard errors for the estimators of all targeted parameters are calculated using a stacked influence curve. Statistical inference for the vector of target parameters is based on this multivariate normal distribution to assess the uncertainty of the estimator. The results are expressed as relative risk and odds ratio (OR), together with their 95% confidence intervals, and the corresponding *P*-values. Continuous variables are presented as median (IQR).

All analyses were performed using R 2.15.1 statistical software running on a Mac OsX platform (SuperLearner and tmle packages, The R Foundation for Statistical Computing, Vienna, Austria).

## Results

### Patients’ characteristics

Characteristics of patients are presented in Table 1: 223 consecutive patients were screened. Among them, 21 were excluded (9 because of RRT before surgery, 4 did not undergo surgery, 5 died during surgery, 3 had incomplete files) and 202 patients were included with a median age of 42 (28 to 59) years. Male patients numbered 134 (66.3%): 19 (9.4%) patients had a previous history of IE.

The median time between onset of symptoms and diagnosis of IE was 15 days (2 to 44). Eighteen patients (9.1%) were in shock prior to surgery, including three (1.5%) who had an episode of circulatory arrest. Seventy-nine patients (39.1%) had symptoms of decompensated heart failure (NYHA functional class III or IV). Eighty-three patients (41.1%) had systemic embolism prior to surgery. The median hospital and ICU lengths of stay were of 34 (20 to 55) days, and 4 (2 to 8) days respectively. The total duration of mechanical ventilation ranged from 1 to 46 days, with a median of 1 (1 to 3) days.

### Surgery

Emergency surgery was required in 27 (13.4%) patients. The median time between diagnosis and surgery was 12 days (3 to 32). Surgery involved the aortic valve in 104 (51.5%) patients, mitral valve in 103 (51%), pulmonary valve in 3 (1.5%), and tricuspid valve in 20 (9.9%) patients. Thirty-five (17.3%) patients had prosthetic valves, and 28 (13.9%) had surgery involving multiple valvular sites. A pacemaker infection was diagnosed in 19 (9.4%) patients. There were 34 (16.8%) mechanical valves and 112 (55.4%) bioprosthetic valves implanted. Valvular plasties were performed in 66 patients (32.7%). One patient (0.5%) had a concomitant coronary artery bypass graft, and fourteen (6.9%) underwent ascending aorta replacement using a prosthetic tube. Median duration of extracorporeal circulation was 118 (85 to 163) minutes, and median duration of aortic cross-clamping was 89 (65 to 121) minutes. Circulatory arrest was necessary in five (2.5%) patients. A second operation (or more) was necessary in 29 (14.4%) patients. The main reasons were: tamponnade (n = 12, 41.4%), valve dysfunction (n = 6, 20.7%) and mediastinitis (n = 4, 13.8%).

### Microbiology

The causative pathogens are presented in Table 2. The three most frequently involved species were: streptococci (n = 84, 41.6%), staphylococci (n = 56, 27.8%) and enterococci (n = 18, 8.9%). Blood cultures were positive in 155 (76.7%) patients.

**Table 2:** Microbiology

	**All patients (n = 202)**	**No post-operative AKI (n = 82)**	**Post-operative AKI (n = 120)**	***P*-value**
***Streptococci***	84 (41.6)	40 (48.8)	44 (36.7)	0.40
*Streptococcus bovis/gallolyticus*	14 (6.9)	5 (6.1)	9 (7.5)	NA
Oral streptococci	36 (17.8)	19 (23.2)	17 (14.2)	NA
Pyogenic streptococci^a^	15 (7.4)	8 (9.8)	7 (5.8)	NA
Others *Streptococcaceae*^b^	19 (9.4)	8 (9.8)	11 (9.2)	NA
***Staphylococci***	56 (27.8)	14 (17.1)	42 (35.0)	0.008
*Staphylococcus aureus*	38 (18.8)	9 (11.0)	29 (24.2)	0.03
Coagulase-negative staphylococci	18 (8.9)	5 (6.1)	13 (10.8)	0.16
***Enterococcus faecalis***	18 (8.9)	6 (7.3)	12 (10.0)	0.68
**HACEK group**	10 (4.9)	6 (7.3)	4 (3.3)	0.66
***Bartonella *** **species**	10 (4.9)	6 (7.3)	4 (3.3)	0.34
***Coxiella burnetii***	3 (1.5)	1 (1.2)	2 (1.7)	0.99
**Others microorganisms**^**c**^	18 (8.9)	9 (11.0)	9 (7.5)	0.55
**Negative culture bacteria **^**d**^	3 (1.5)	0 (0)	3 (2.5)	0.39

^a^Beta-hemolytic streptococci (group A,B,C and G); ^b^Others streptococci: *Streptococcus pneumoniae* (n = 6), *Peptostreptocccus* (n = 2), *Abiotrophia defectiva* (n = 1), *Gemella* species (n = 3), *Streptococcus acidominimus* (n = 1)*, Streptococcus anginosus* (n = 1)*, Streptococcus deficiens* (n = 1)*, Streptococcus spp* (n = 4); ^c^Others micororganisms: *Serratia marcescens* (n = 1), *Klebsiella pneumoniae* (n = 1), *Proprionobacterium* species (n = 5), *Candida* species (n = 1), *Aspergillus* species (n = 1), *Campylobacter* species (n = 1); others (n = 8); ^d^For the three patients with negative cultures, the direct examination revealed Gram-positive Cocci; HACEK, *Haemophilus sp, Actinobacillus actinomycetemcomitans, Cardiobacterium hominis, Eikenella corrodens, Kingella kingae;* NA, *not applicable.*

### Renal function and prognosis

**Figure 1:**
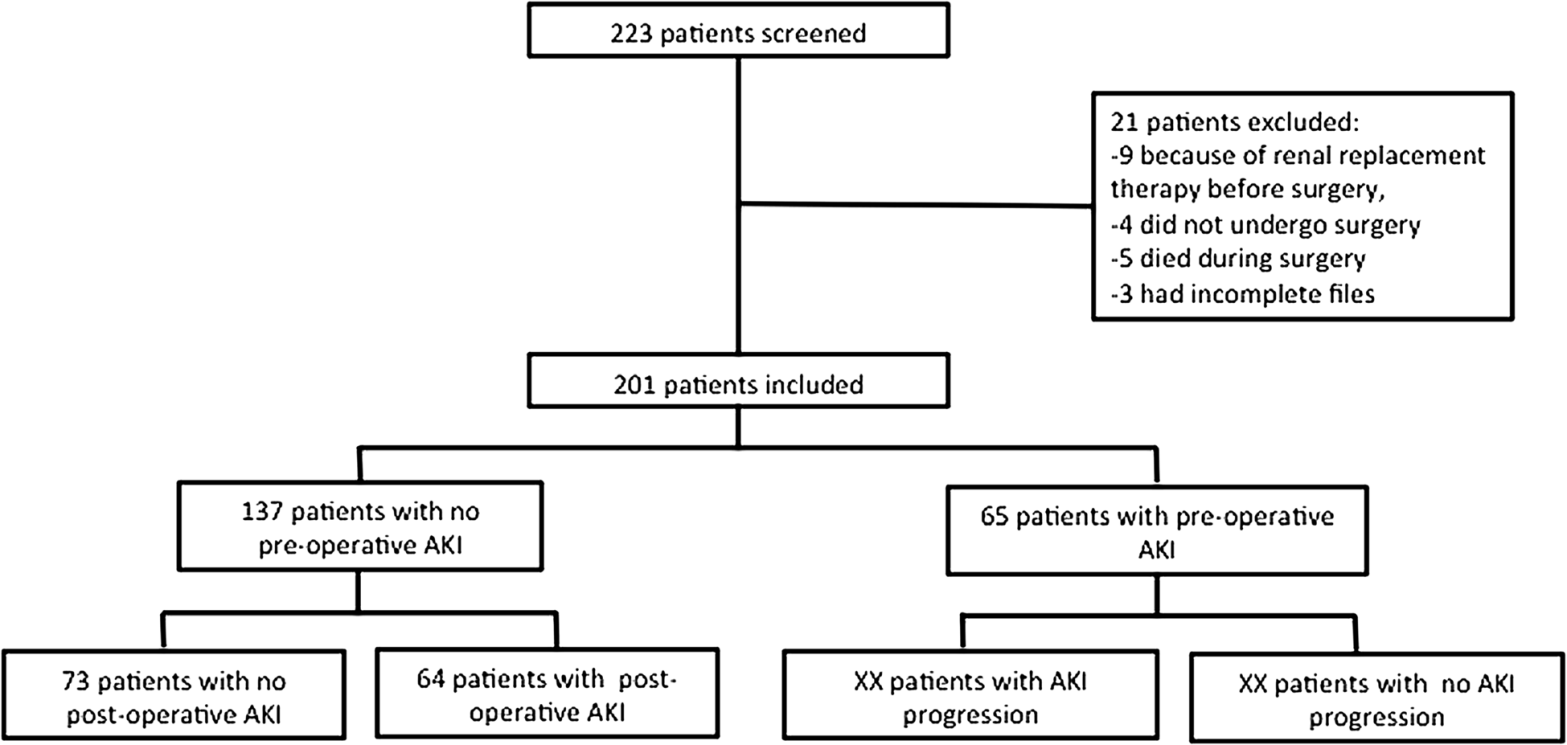
**Flow chart of patients.** AKI, acute kidney injury.

**Figure 2:**
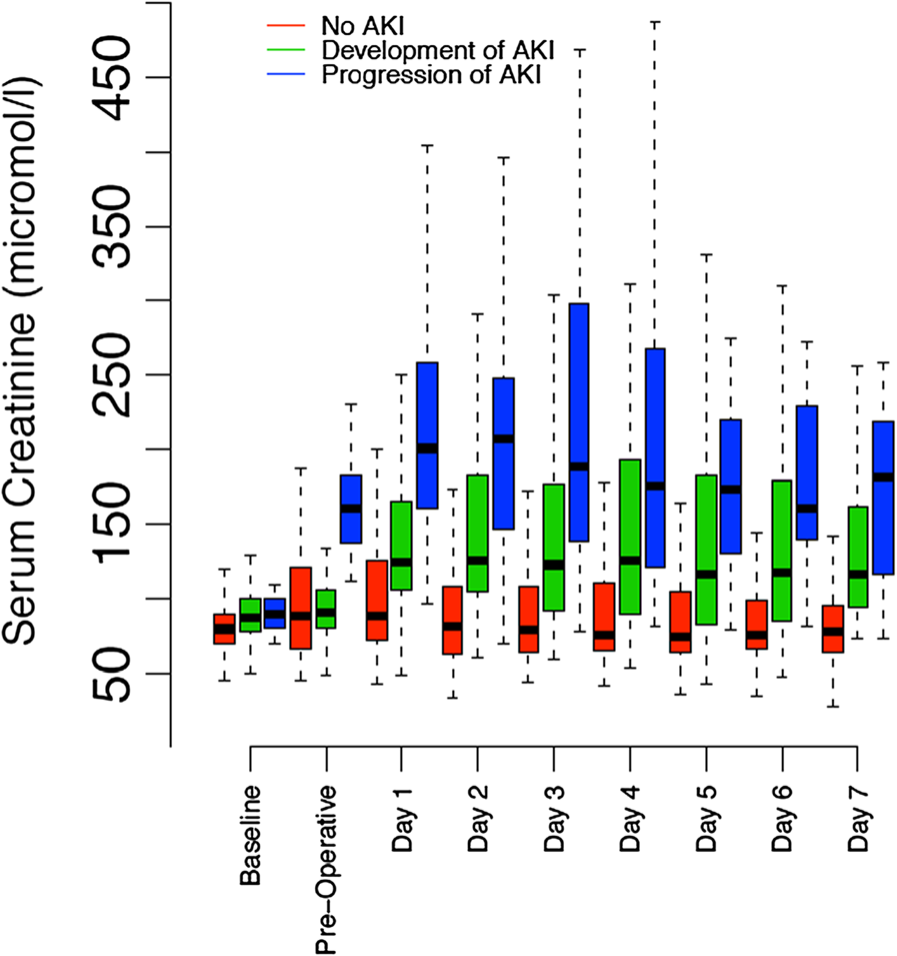
**Evolution of serum creatinine during the peri-operative period.** AKI, acute kidney injury.

Sixty-five patients (32.2%) presented with AKI before surgery, and 137(67.8%) had normal renal function before surgery. Of the 65 patients with pre-operative AKI, 52 (80%) were in AKIN stage 1, 9 (13.8%) in stage 2 and 4 (6.2%) in stage 3. A flow chart depicting the evaluation of renal function throughout the peri-operative period is provided in Figure 1. The evolution of serum creatinine in the peri-operative period is separately described in Figure 2.

A total of 120 (59.4%) patients met the criteria for AKI in the post-operative period: 64 (46.7%) of the 137 patients without pre-operative AKI and 56 (86.1%) of the 65 patients with pre-operative AKI. Among the 65 patients with pre-operative AKI, 9 (13.8%) recovered during the 7 days following surgery, 29 (44.6%) had no AKI progression, and 27 (41.6%) experienced AKI progression (that is, worsening in their AKI stage).

A total of 20 patients (9.9%) required RRT during the post-operative ICU stay. The delay between ICU admission and the initiation of RRT was 1.5 (1 to 3) days.

#### Association between acute kidney injury and patients’ outcomes

**Figure 3:**
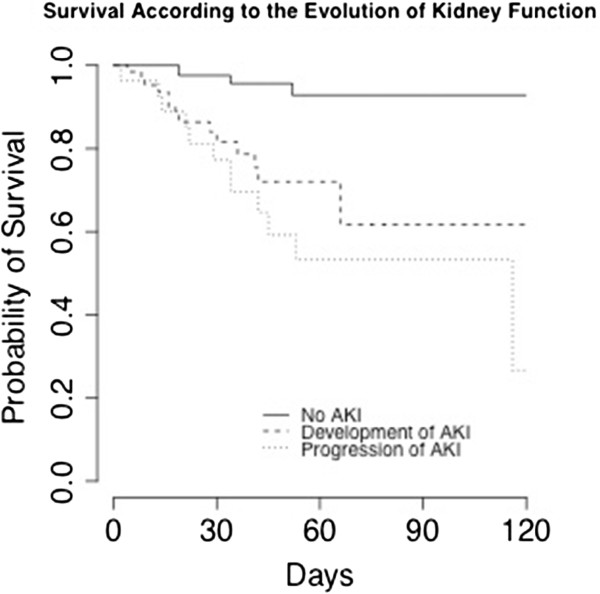
**Survival according to the evolution of kidney function.** AKI, acute kidney injury.

Thirty patients (14.8%) died during their hospital stay. Mortality was associated with the presence/absence of AKI. The association was not statistically significant when considering pre-operative AKI only: 16 (11.7%) patients without pre-operative AKI died versus 14 (21.5%) patients with pre-operative AKI (*P* = 0.09 for univariate analysis). However, the association between post-operative AKI and mortality was statistically significant: 2 (2.4%) patients without post-operative AKI died, and 28 (23.3%) patients with post-operative AKI died during their hospital stay (*P* <0.001 for univariate analysis). AKI stage was also associated with mortality: 18 (52.9%) patients who experienced AKI stage 3 during the peri-operative period died, but only 12 (7.1%) of the patients who did not reach stage 3 died during their hospital stay (*P* <0.001 for univariate analysis). The course of AKI during the peri-operative period was also strongly associated with mortality: among the patients whose renal function improved, only one (6.2%) died, three (3.2%) of the patients whose renal function remained stable died, and 26 (28.6%) of those whose renal function deteriorated died during their hospital stay (*P* <0.001 for univariate analysis) (Figure 3).

The AKI stage was also significantly associated with the duration of mechanical ventilation (*P* <0.001) and the ICU length of stay (*P* <0.001), but not with the hospital length of stay (*P* = 0.08).

#### Prediction of renal function worsening after surgery

The first step was to predict post-operative AKI during the seven days following surgery. Of the candidate algorithm considered for prediction, stepwise regression AIC had the lowest cross-validated mean squared prediction error. The cross-validated AUROC for this algorithm was 0.757 (95% CI 0.689, 0.826) (Additional file 1). The super learner weighted estimator achieved an estimated AUROC of 0.760 (95% CI 0.694, 0.826).

Variable importance measure estimated using TMLE identified the following risk factors for post-operative AKI (Table 3): multiple surgery (OR 4.16, 95% CI 2.98, 5.80, *P* <0.001), pre-operative anemia as defined by a baseline hemoglobin level <10 g/dl (OR 1.89, 95% CI 1.34, 2.66, *P* <0.001), transfusion requirement during surgery (OR 2.38, 95% CI 1.55, 3.63, *P* <0.001), the use of a nephrotoxic agent: vancomycin (OR 2.63, 95% CI 2.07, 3.34, *P* <0.001), aminoglycoside (OR 1.44, 95% CI 1.13, 1.83, *P* = 0.004) or contrast iodine (OR 1.70, 95% CI 1.37, 2.12, *P* <0.001); and the interaction between vancomycin and aminoglycoside (OR 2.62, 95% CI 2.08, 3.31, *P* <0.001). On the contrary, age over 65 y was found to protect for experiencing the outcome (OR 0.41, 95% CI 0.30, 0.57, *P* <0.001). However, when comparing middle-aged patients (40 to 75 y) to the elderly (>75 y), the effect of age was no longer significant (OR 0.80, 95% CI 0.55, 1.18, *P* = 0.26).

**Table 3:** Factors associated with impairment in renal function after surgery

	**RR**	**95% CI**	***P*-value**	**OR**	**95% CI**	***P*-value**
**Multiple surgery**	1.83	1.54, 2.18	<0.001	4.16	2.98, 5.80	<0.001
**Vancomycin administration**	1.57	1.37, 1.81	<0.001	2.63	2.07, 3.34	<0.001
**Aminoglycoside administration**	1.23	1.07, 1.41	0.004	1.44	1.13, 1.83	0.004
**Vancomycin:aminoglycoside interaction**	1.54	1.34, 1.76	<0.001	2.62	2.08, 3.31	<0.001
**Contrast agent**	1.33	1.18, 1.51	<0.001	1.70	1.37, 2.12	<0.001
**Transfusion**	1.62	1.27, 2.07	<0.001	2.38	1.55, 3.63	<0.001
**Pre-operative hemoglobin (<10 g/dl)**	1.39	1.15, 1.67	0.001	1.89	1.34, 2.66	<0.001
**Age (>65 y)**	0.57	0.47, 0.70	<0.001	0.41	0.30, 0.57	<0.001

The variable importance measure for the risk factors associated with post-operative renal function impairment was performed after adjusting for all other covariates. RR, relative risk, OR, odds ratio.

## Discussion

The present study aimed to investigate the incidence, risk factors and prognosis associated with the post-operative AKI during the seven days following cardiac surgery for IE. We identified multiple surgery, pre-operative anemia, transfusion requirement during surgery and the use of nephrotoxic agents within 48 hours before surgery, to be significant risk factors.

In this study we restricted our analysis to patients with IE requiring cardiac surgery, for several reasons. First, the role of surgery in the treatment of IE has been emphasized with recent studies, suggesting that early surgical management could decrease the risk of stroke and death [1,19]. Second, most patients treated medically have contraindications to surgery, such as severe comorbid conditions or poor performance status. In the latter patients, prior conditions may per se strongly increase the risk of mortality or morbidities [20]. Third, although risk factors for AKI following cardiac surgery have been previously studied, patients with IE are likely to differ because of the ongoing inflammatory and infectious processes. Even though surgery aims to control the infectious process, we hypothesized that the accumulation of injuries, such as infection, systemic inflammation related to the cardiopulmonary bypass or the use of nephrotoxic agents, further increase the risk of renal failure after surgery in such patients. We finally confirmed that patients with IE have a high risk of post-operative AKI following cardiac surgery [2-4].

We identified pre-operative anemia as a risk factor for post-operative AKI. Our findings are in line with the study from Karkouti *et al*. who observed a relationship between pre-operative hemoglobin and AKI after cardiopulmonary bypass [21,22]. The reasons for such an association are likely multifactorial. Several experimental studies have stressed the susceptibility of the kidney to anemia, and the occurrence of renal hypoxia after decrease of hemoglobin level due to maintenance of high oxygen consumption and intrarenal oxygen shunting [23]. In a rat model where renal oxygen tension was altered by hemodilution despite normal arterial blood pressure [24], a specific contribution of anemia to kidney damage through oxidative stress has been proposed [25]. Moreover, we found that red blood cell (RBC) transfusion per se was also a risk factor. Several authors have previously identified the negative impact of RBC transfusion on renal function after cardiac surgery. One of the reasons could be the inability of RBC transfusion to restore adequate microcirculatory oxygenation because of the multiple morphological and functional changes (less deformability, depletion of 2,3-diphosphoglycerate, inflammation, decrease of bioavailability of nitric oxide with liberation of free hemoglobin) occurring during blood storage.

Peri-operative administration of nephrotoxic agents, such as vancomycin, aminoglycosides or contrast iodine, was also found to be a risk factor. Furthermore, the interaction between vancomycin and aminoglycosides was also found to be a significant risk factor. This would suggest that these two drugs, when administrated together, might have potentialized nephrotoxicity. Vancomycin-induced nephrotoxicity has been much debated. Vancomycin has been described as nephrotoxic in patients with IE, in critically ill patients, especially after prolonged administration. High values for serum trough concentrations of vancomycin have been associated with an increased risk of AKI [26,27]. However maintenance of higher trough concentration is often required in serious methicillin-resistant *Staphylococcus aureus* infections because of the high minimum inhibitory concentration. Alternative strategies using less nephrotoxic antibiotics, such as daptomycin, certainly merits further evaluation in patients undergoing operation for IE [28,29]. Although aminoglycosides are well-known nephrotoxic agents, they have been scarcely studied in patients with IE and their indication remains debated [30]. Buchholtz *et al*. have specifically explored the nephrotoxic effects of aminoglycosides in patients with IE and observed that worsening of renal function correlated with the duration of gentamicin treatment [31]. Pooling together the results of four randomized controlled trials that included patients with IE, the relative risk of nephrotoxicity was 2.22 (95% CI 1.11, 4.35) in patients treated with aminoglycosides [32]. Consistently, in a recent randomized controlled trial, Fowler *et al*. reported fewer episodes of renal failure in patients treated with daptomycin compared with patients receiving aminoglycosides for IE (11 versus 26% respectively) [28]. Moreover, regarding risk associated with iodine contrast in our series, the benefit of contrast-enhanced computer tomography or angiography pre-operatively should be balanced with the risk to renal function. Finally, we identified multiple surgery as another risk factor for post-operative worsening of renal function. Multiple surgery exposes the kidney to repeated factors of aggression, including hemodynamic instability, renal venous congestion when tamponnade occurs, inflammatory response to cardiopulmonary bypass, anemia and RBC transfusions. We found that receiving several nephrotoxic agents in the 48 hours before surgery was an important risk factor for post-operative AKI. We realize that these agents are sometimes needed, but we highlight that this period is of high risk for the kidney and these agents should be avoided as much as possible during this period.

In contrast with previous studies [33], we found no association between the causative pathogen and the risk of worsening in renal function in our cohort. Several studies have linked Staphylococcus species infections to poor outcome in patients with IE. Several reasons can be proposed to explain such a difference. First, our results might suggest that the causal relationship between Staphylococcus-related IE and kidney injury needs to be questioned. Indeed, the association between Staphylococcus species and AKI may be attributable - at least in part - to the fact that patient with Staphylococcus-related IE was more likely to be treated with vancomycin and aminoglycosides. Second, only patients undergoing surgery were included in our study, therefore, excluding patients with the most advanced comorbidities, older age or poor performance status, who were considered too ill or too old to benefit from surgery. Unexpectedly, age over 65 y was found to be associated with less impairment in renal function in the post-operative period. The fact that age was found to be protective in our cohort further supports a selection of patients prior to surgery. Indeed, while the majority of the young patients were considered suitable for surgery, only the healthiest elderly were selected to undergo surgical treatment. Such a protective effect of aging could also be explained by the presence of young patients in the cohort, in whom the prevalence of post-operative degradation in renal function was very high. This interpretation was supported by the fact that when comparing middle-aged patients (40 to 75 y) to the elderly (>75 y), the effect of age was no longer significant (OR 0.80, 95% CI 0.55, 1.18, *P* = 0.26).

Although AKI has been associated with a higher risk of mortality in patients with IE, the timing and different contributing factors of AKI have not been clearly explored so far. In a Spanish multicenter observational study of 705 patients with left-sided IE, using multivariate analysis, Gálvez-Acebal *et al*. reported AKI to be associated with mortality [34]. In our series, we found that post-operative AKI was clearly associated with in-hospital mortality, whereas the association between pre-operative AKI (excluding patients receiving pre-operative RRT) and mortality was not significant. This suggests the importance of developing and evaluating perioperative strategies to prevent the occurrence of post-operative AKI. Interestingly, we observed a sharp difference in mortality between patients reaching stage 3 AKI and patients with stage 1 or 2, suggesting that all forms of AKI should not be considered equal in their severity in this setting. Although we only observed a statistical association between AKI progression and mortality, we cannot exclude a lack of power in our cohort to show such an association with pre-operative AKI.

The statistical analysis used some innovative tools such as SuperLearner [35] for prediction and TMLE [16,17] for estimation. The idea behind super learning is to optimize the prediction performances, accepting the fact that we do not know anything about the true shape of the underlying data distribution, so that every kind of parametric regression model would be biased. SuperLearner allows us to use a large library of candidate regression algorithms, parametric of data-adaptive, to honestly evaluate their prediction performance, and to build a new, tailored algorithm that is a combination of the best candidates. As expected from theory, we found that SuperLearner outperformed each candidate algorithm included in its library. From such results, we expect SuperLearner to do the best possible job to estimate the overall probability distribution of the outcome in our dataset. However, when looking at risk factors, we do not really care about the whole probability distribution of the outcome. In fact, we do care about a far less dimensional object, which is the distribution of the outcome given the level of a given potential risk factor. Our efforts should then focus on optimizing the bias/variance tradeoff for this object rather than for the whole outcome probability distribution. The TMLE aims at targeting our estimation in a way that the optimal bias/variance tradeoff is achieved for the parameter of interest.

Our study has several limitations. First, it was performed in a tertiary center with wide experience in medical and surgical treatment of patients with IE, potentially limiting the external validity. Second, our definition of AKI was based on serum creatinine level and did not include urine output. Third, we only have limited information on transfusion requirements. We were only able to evaluate the risk associated with peri-operative transfusion, but not the amount of blood transfused, nor the risk associated with pre- or post-operative transfusion. Fourth, the results apply to a selected population of patients suitable for surgery. Finally, despite the SuperLearner procedure, which is intended to optimize the prediction, our predictive performance was in fact limited, with an AUROC of 0.760 (95% CI 0.694, 0.826) for the SuperLearner weighted algorithm. Improved predictive performance might first be achieved by expanding the library of candidate algorithms, as the SuperLearner is at least as good as the best of its library. Hence, it remains to be investigated if a more aggressive library will result in further improvements. If not, one should consider expanding the set of predictive variables considered, including, potentially, specific kidney biomarkers.

## Conclusions

This study adds evidence to the current literature suggesting that post-operative AKI following cardiac surgery with cardiopulmonary bypass results from additive hits to the kidney. It also identified several modifiable risk factors of post-operative AKI such as treatment with vancomycin or aminoglycosides or pre-operative anemia. Development of post-operative AKI was associated with in-hospital mortality. Prevention or correction of these risk factors may improve outcome in patients with IE undergoing cardiac surgery.

## Key messages

•>50% patients undergoing cardiac surgery for IE develop post-operative AKI

•We identified six risk factors for post-operative AKI, most being treatment-related, including vancomycine and aminoglycoside administration, and contrast agent.

•Pre-operative anemia was associated with an increased risk of post-operative AKI

•Post-operative AKI was associated with an increased risk of death.

## Abbreviations

AIC: Akaike information criterion; AKI: Acute kidney injury; AKIN: Acute kidney injury network; AUROC: Area under the receiver operating curve; CPB: Cardiopulmonary bypass; COPD: Chronic obstructive pulmonary disease; GFR: Glomerular filtration rate; IE: Infective endocarditis; LVEF: Left ventricular ejection fraction; MDRD: Modification of diet in renal disease; NYHA: New York Heart Association; OR: Odds ratio; RBC: Red blood cell; RR: Relative risk; RRT: Renal replacement therapy; TMLE: Targeted maximum likelihood estimation.

## Competing interests

JLM declares having received research grants from Novartis, Aventis, MSD; speaker fees from Pfizer, Novartis, Aventis, AstraZeneca, GSK; travel grants from Pfizer, Novartis, Janssen, Aventis, Wyeth, Astellas and is scientific adviser for Aventis and AstraZeneca.

Other authors declare that they have no competing interests.

## Authors’ contributions

ML designed the study, contributed to acquisition, analysis and interpretation of data; drafted the manuscript and has given final approval of this version; RP contributed to analysis and interpretation of data; drafted the manuscript and has given final approval of this version; AR contributed to acquisition, analysis and interpretation of data and has given final approval of this version; MLP revised the manuscript critically for important intellectual content and has given final approval of this version; MVL revised the manuscript critically for important intellectual content and has given final approval of this version; JNF revised the manuscript critically for important intellectual content and has given final approval of this version; MPFG revised the manuscript critically for important intellectual content and has given final approval of this version; IP revised the manuscript critically for important intellectual content; DS revised the manuscript critically for important intellectual content and has given final approval of this version; BC revised the manuscript critically for important intellectual content and has given final approval of this version; JLM designed the study, contributed to acquisition, analysis and interpretation of data; drafted the manuscript and has given final approval of this version. All authors read and approved the final manuscript.

## Supplementary Material

Additional file 1 **(A) *Super Learner*-based cross-validated risk.** MSE, cross validated mean squared error; AUROC, cross-validated Area Under the Receiver Operating Curve. **(B)** Results of the variable importance measures using targeted maximum likelihood estimation (TMLE) for the candidate risk factors for renal function worsening. CPB, cardiopulmonary bypass; RR, relative risk; OR, odds ratio; IE, infectious endocarditis. Click here for file
