# Supplementary material for: Incidence, risk factors and prediction of post-operative acute kidney injury following cardiac surgery for active infective endocarditis: an observational study
Source: Crit Care. 2013 Oct 4;17(5):R220. doi: 10.1186/cc13041 (PMC4056899; doi:10.1186/cc13041)
Supplement: Additional file 1 — (A) Super Learner-based cross-validated risk. MSE, cross validated mean squared error; AUROC, cross-validated Area Under the Receiver Operating Curve. (B) Results of the variable importance measures using targeted maximum likelihood estimation (TMLE) for the candidate risk factors for renal function worsening. CPB, cardiopulmonary bypass; RR, relative risk; OR, odds ratio; IE, infectious endocarditis. [file cc13041-S1.doc]

**Appendix A. *Super Learner* based cross-validated risk.** MSE : cross validated Mean Squared Error, SD : standard deviation, AUROC : cross validated Area Under the Receiver Operating Curve, 95%CI: 95% confidence interval.

|  | **MSE** | **SD** | **AUROC** | **95%CI** |
| --- | --- | --- | --- | --- |
| **Super Learner** | 0.208 | 0.013 | 0.760 | 0.694 - 0.826 |
| **Logistic Regression** | 0.246 | 0.022 | 0.718 | 0.647 - 0.789 |
| **Generalized Additive Model** | 0.245 | 0.022 | 0.722 | 0.651 - 0.794 |
| **Stepwise Regression Forward** | 0.245 | 0.020 | 0.709 | 0.637 - 0.781 |
| **Stepwise Regression AIC** | 0.205 | 0.016 | 0.757 | 0.689 -0.826 |
| **Neural Network** | 0.224 | 0.011 | 0.631 | 0.513 - 0.748 |
| **Random Forest** | 0.207 | 0.012 | 0.749 | 0.680 - 0.817 |
| **Boostrap Aggregation of Trees** | 0.209 | 0.012 | 0.753 | 0.684 - 0.822 |
| **Gradient Boosting** | 0.206 | 0.012 | 0.752 | 0.683 - 0.820 |
| **Polynomial Spline Regression** | 0.210 | 0.017 | 0.746 | 0.677 - 0.816 |
| **Bayesian Linear Model** | 0.225 | 0.019 | 0.734 | 0.664 - 0.804 |
| **Elastic Net** | 0.208 | 0.011 | 0.749 | 0.680 -0.817 |

**Appendix B. Results of the variable importance measures using TMLE for the candidate risk factors for renal function worsening.** CPB: cardiopulmonary bypass; TMLE: targeted maximum likelihood estimation; RR: relative risk; 95%CI: 95% confidence interval; OR: odds ratio; IE: infectious endocarditis.

|  | **RR** | **95%CI** | **p value** | **OR** | **95%CI** | **p value** |
| --- | --- | --- | --- | --- | --- | --- |
| **Multiple Surgery** | 1.83 | 1.54-2.18 | <0.001 | 4.16 | 2.98-5.80 | <0.001 |
| **Vancomycin administration** | 1.57 | 1.37-1.81 | <0.001 | 2.63 | 2.07-3.34 | <0.001 |
| **Aminoglycoside administration** | 1.23 | 1.07-1.41 | 0.004 | 1.44 | 1.13-1.83 | 0.004 |
| **Vancomycin : Aminoglycoside** | 1.54 | 1.34-1.76 | <0.001 | 2.62 | 2.08-3.31 | <0.001 |
| **Contrast agent** | 1.33 | 1.18-1.51 | <0.001 | 1.70 | 1.37-2.12 | <0.001 |
| **Transfusion** | 1.62 | 1.27-2.07 | <0.001 | 2.38 | 1.55-3.63 | <0.001 |
| **Hemoglobin (<10 g/dl)** | 1.39 | 1.15-1.67 | 0.001 | 1.89 | 1.34-2.66 | <0.001 |
| **Age (>65 y/o)** | 0.57 | 0.47-0.70 | <0.001 | 0.41 | 0.30-0.57 | <0.001 |
| **Diabetes Mellitus** | 1.13 | 0.99-1.27 | 0.06 | 1.25 | 0.99-1.58 | 0.05 |
| **Chronic Hypertension** | 0.93 | 0.80-1.09 | 0.40 | 0.88 | 0.67-1.17 | 0.39 |
| **Chronic Liver Disease** | 0.96 | 0.81-1.15 | 0.69 | 0.94 | 0.68-1.29 | 0.69 |
| **Baseline Creatinine** | 0.87 | 0.71-1.06 | 0.17 | 0.78 | 0.56-1.10 | 0.16 |
| **History of IE** | 0.99 | 0.71-1.39 | 0.97 | 0.99 | 0.58-1.68 | 0.97 |
| **Preoperative Shock** | 1.02 | 0.86-1.22 | 0.78 | 1.05 | 0.75-1.47 | 0.79 |
| **Native vs. prosthetic valve** | 1.06 | 0.96-1.17 | 0.22 | 1.11 | 0.93-1.32 | 0.23 |
| ***Streptococcus* species vs. others** | 1.12 | 0.97-1.30 | 0.13 | 0.91 | 0.71-1.16 | 0.44 |
| ***Staphylococcus* species vs. others** | 1.12 | 0.97-1.30 | 0.13 | 1.23 | 0.94-1.60 | 0.13 |
| **Positive Blood Culture** | 1.02 | 0.86-1.19 | 0.84 | 1.03 | 0.76-1.40 | 0.84 |
| **Emergency surgery** | 1.22 | 1.00-1.48 | 0.05 | 1.50 | 0.98-2.29 | 0.06 |
| **CPB duration** | 1.23 | 0.99-1.53 | 0.06 | 1.50 | 0.97-2.30 | 0.07 |
| **Aortic clamping duration** | 1.04 | 0.89-1.22 | 0.62 | 1.07 | 0.81-1.42 | 0.62 |
| **Maximum Troponin** | 1.06 | 0.93-1.20 | 0.40 | 1.11 | 0.88 -1.40 | 0.40 |
